# The Effect of Glucose Levels Prior to Hematopoietic Cell Transplantation on Post-Transplant Complications and Health Resource Utilization 

**Published:** 2019-07-01

**Authors:** Amir Steinberg, Janet H. Van Cleave, Anish B. Parikh, Erin Moshier, Meng Ru, Molly Lawson, Douglas Marks, Antoinette Montelibano, Amanda Philpott, Kourtney Garner, Marilyn J. Hammer

**Affiliations:** 1 Division of Hematology/Oncology, Tisch Cancer Institute, Icahn School of Medicine Mount Sinai, New York, NY, USA; 2 Department of Nursing, Mount Sinai Hospital, Icahn School of Medicine at Mount Sinai, New York, NY, USA; 3 New York University Rory Meyers College of Nursing, New York, NY, USA; 4 Department of Population Health Science and Policy, Icahn School of Medicine at Mount Sinai, New York, NY, USA; 5 Division of Hematology/Oncology, Irving Comprehensive Cancer Center, Columbia University Medical Center, New York, NY, USA

**Keywords:** Glucose, Hyperglycemia, Patient readmission, Bone marrow transplant, Infection, Health resource utilization

## Abstract

**Background: **Abnormal blood glucose (BG) levels during hematopoietic cell transplantation (HCT) are associated with increased infections, delayed engraftment, and prolonged hospitalization, though little is known about these associations.

**Materials and Methods: **We retrospectively evaluated mean BG levels in the week prior to HCT and subsequent outcomes for 852 HCTs at our hospital from 1/2009 – 12/2013 pertaining to 745 patients. Outcomes included infections (pneumonia, *C. difficile*, positive cultures, administration of antimicrobials, or neutropenic fever), time-to-engraftment (TTE), and quality indicators (30- and 90-day readmission rates [RR] and median length-of-stay [LOS]).

**Results:** 404 patients met the criteria for involvement in this study. The population was 55% male and was racially and ethnically mixed (White 38%, African American 23%, Hispanic 6%, Asian 7%, Other 21%). Mean age was 57+14 years. Significantly more patients in Group 2 were diagnosed with pneumonia (19%) compared with the Group 1 (7%) and Group 3 (10%) [p=.0054]. Patients in Group 2 also had significantly longer median LOS: Group 1-23 days, Group 2-26 days, Group 3-22 days [p = .0157]. No significant differences were noted in terms of the other infectious complications or in time-to-engraftment or readmissions.

**Conclusion: **Pre-HCT BG trends may be a prognostic biomarker for adverse outcomes, and thus can help improve quality of care for HCT patients.

## Introduction

 Public policy has increasingly emphasized enhancing healthcare quality while reducing associated costs^1^. Due to its inherent complexity, cancer care represents a significant proportion of total healthcare expenditures, with costs rising each year. The cost of cancer care alone was estimated to be $125 billion in the US in 2010, and is projected to reach $158 billion by 2020^2^. Investigation of factors contributing to adverse events and outcomes can inform early interventions to help improve the care of cancer patients while mitigating resource utilization. 

There is growing evidence that abnormal blood glucose levels, termed *malglycemia,* contribute to adverse outcomes in cancer patients^3^. Defined as hyperglycemia (blood glucose [BG] > 126 mg/dL), hypoglycemia (BG < 70 mg/dL), or increased glycemic variability (standard deviation [SD] between BG measurements of > 29 mg/dL)^3^, malglycemia is most common among diabetics, however, is not isolated to this group. Diabetes prevalence among cancer patients in the US is as high as 18%, versus 9.4% in the general population^4^^,^^5^. The American Cancer Society and American Diabetes Association issued a joint statement in 2010 detailing strong epidemiological evidence for a link between diabetes and certain malignancies, including cancers of the liver, pancreas, endometrium, colon, rectum, breast, and bladder^6^.

Hyperglycemia is the most common malglycemic state and is linked to adverse health outcomes via inflammation. Hyperglycemia triggers the release of cortisol, epinephrine, norepinephrine, glucagon, and growth hormone, leading to increased insulin resistance, lipolysis, gluconeogenesis, glycogenolysis, and decreased insulin secretion, thereby further promoting hyperglycemia^7^. Concurrently, hyperglycemia increases cytosolic calcium levels which induces mitochondrial fragmentation creating increased reactive oxygen species (ROS), the overabundance of which causes oxidative stress^8^. This alters cell surface and cytosol pattern recognition receptor (PRR) function within innate immune cells that normally detect pathogen-associated molecular patterns^9^^,^^10^. The toll-like receptor PRRs activated by oxidative stress trigger a signaling cascade that evokes activation of NF-kB, STAT3, and HIFI-, all transcription factors that induce cytokine, chemokine, and prostaglandin expression^11^. In particular, the proinflammatory cytokines IL-6, TNF-, and IFN- are expressed at pathologically high levels^11^^-^^13^. Other cytokine mediators of inflammation (IL-8, IL-10, IL-18) may also be involved in this pathway. Hyperglycemia also causes the formation of superoxide instead of utilizing glucose molecules through the tricarboxylic acid cycle, one of the mechanisms for ATP production^14^^,^^15^. The sequelae of these events compromises natural WBC function, including complement fixation, cell adherence, chemotaxis, phagocytosis, and direct killing of infectious microorganisms, thereby allowing pathogens to thrive^16^. Additionally, in cancer patients, the already compromised immune system allows the malignancy to progress, further increasing cancer-related inflammation^9^.

Less is known about the impact of malglycemia during hematopoietic cell transplantation (HCT). HCT recipients may be prone to malglycemia regardless of pre-existing diabetes due to various other factors, including older age^5^^,^^17^,high body mass index^18^, nutritional imbalances^16^^,^^19^^,^^20^, low physical activity levels^21^^,^^22^, high stress levels^16^^,^^23^, use of glucocorticoids^16^^,^^24^^-^^26^, and infections^27^. Preliminary studies have shown that patients who experience malglycemia during HCT may be at increased risk for infection and non-malignancy related death^3^^,^^28^^,^^29^. Malglycemia also appears to affect hematopoeisis, as one study showed impaired hematopoietic progenitor function in mice with prolonged hyperglycemia^30^. Furthermore, glycemic status has also been shown to influence length-of-stay (LOS) in patients receiving total parenteral nutrition (TPN)^31^.

The Centers for Medicare and Medicaid Services (CMS) uses hospital length and readmission rates (RR) as benchmarks for improving care. We hypothesize that patients who experience malglycemia, particularly hyperglycemia, prior to HCT experience more post-HCT adverse events, which then lead to increased resource utilization. Based on prior data described by our group and in the literature, we aimed to study the association between pre-HCT glucose trends and both clinical and quality outcomes. Understanding these potential correlations, which to our knowledge have not been studied before, can help inform interventions to enhance glycemic control in order to improve patient outcomes and overall quality of care in this vulnerable population. 

## MATERIALS AND METHODS

**Study design**

This study consisted of a retrospective review of medical records from Mount Sinai Hospital in New York City. Data from 852 HCTs pertaining to 745 distinct patients were obtained from our institution’s data warehouse and the Center for International Blood and Marrow Transplant Research (CIBMTR). Only patients with > 3 available serum BG measurements during the 7 days prior to HCT between January 1, 2009 and December 31, 2013 were included. Exclusion criteria were absence of BG data the day before HCT (day -1) and patients with BG > 265 mg/dL during this 7-day period. Patients with pre-existing diabetes were not excluded. This project was approved by our Institutional Review Board (IRB) prior to initiation of the study. 

**Patient characteristics**

Data were collected from the databases regarding date of admission, race, ethnicity, gender, diagnosis, type of HCT, receipt of TPN after HCT, and receipt of steroids (prednisone, prednisolone, dexamethasone, methylprednisolone, or budesonide) after HCT. ICD-9 codes were used to identify patients with a history of diabetes (250.x) and graft-versus-host disease (279.5x). Medical records and discharge summaries were manually reviewed to identify patients who received an endocrinology consult. 

**Glycemic measurements**

Serum BG measurements were obtained in one of the two ways. Central laboratory measurements were obtained by phlebotomy staff daily between 0300 and 0600 hours on all patients. Capillary point-of-care measurements were drawn four times daily by nursing staff from patients with pre-existing diabetes as per the medical record or who had elevated BG levels on daily central laboratory measurements. These BG measurements were collected daily until the day of HCT. All glycemic measurements, regardless of method of collection, were verified in the medical record.

**Trajectory modeling**

Group-based trajectory modeling (GBTM) was used to identify groups of patients with similar BG patterns in the 7 days prior to HCT. This approach applies a multinomial model to identify relatively homogenous groups of distinctive trajectories summarized by a set of functions of time. Selection of the optimal number of trajectory groups was guided by four indices: (1) Bayesian information criterion (BIC) with smaller BIC indicating better fit; (2) entropy, a standardized measure  of a patient’s probability of being in the most likely group with values > 0.80 indicating good classification quality; (3) average posterior probability for each group with values > 0.70 indicating good accuracy of classification; and (4) estimated proportion of patients belonging to each trajectory group with recommended values > 0.05^32^. Models were fit using two to seven trajectory groups with a third-order polynomial. Once the optimal number of groups was identified, the order of polynomial models of each group was reduced until the highest order for each group was still significant. Using these criteria, we selected a 3-group quadratic trajectory model.

**Clinical outcomes: infectious complications and time-to-engraftment**

The post-HCT infectious complications that we analyzed were selected based upon prior research showing an association between BG levels and infections^29^. These complications were a diagnosis of pneumonia or *Clostridium difficile* (C. difficile) infection, positive bacterial cultures, receipt of antimicrobials, or occurrence of neutropenic fevers. Potential cases of pneumonia were identified by searching for relevant ICD-9 codes (793.1 and 518.3) and by text-mining radiology reports using the keywords “infiltrate,” “pneumonia,” or “opacity” for all patients. The charts for these patients were then reviewed, and patients were classified as actually having had pneumonia if records indicated that there was a subsequent change in management or if a diagnosis was made by an infectious disease or HCT clinician. *C. difficile* infection was defined as having positive EIA toxin A/B testing before updated laboratory testing in 2012, or positive toxin B PCR after. Positive bacterial cultures referred to one of the following: urine containing >100 000 colony forming units of bacteria, one positive blood culture (or two positive blood cultures in the case of coagulase-negative staphylococci), or an organism identified in sputum or bronchial washings. Antimicrobial treatment referred to the receipt of either vancomycin, cefepime, imipenem, aztreonam, metronidazole, linezolid, daptomycin, ambisome, posaconazole, caspofungin, voriconazole, meropenem, piperacillin-tazobactam, gentamicin, amikacin, or tobramycin. Neutropenic fevers were defined as a temperature > 37.9C when absolute neutrophil count (ANC) was less than 500. All data were verified with the individual medical records. 

The association between BG levels and time-to-engraftment was analyzed as delayed engraftment, and was hypothesized to lead to more health resource use. Engraftment was defined as ANC 1 000 cells/mm^3^ per CIBMTR criteria. Data regarding date of engraftment were obtained from the CIBMTR database and were verified in the medical record. 

**Quality outcomes: health resource utilization**

Utilization of health resources has historically been measured in the volume of units of physician and hospital services rendered^33^. More recently, studies of health services have included patterns of readmissions rates (RR) as a measure of quality of care after discharge^34^. To capture both volume of services and quality of care, the health resource utilization outcome variables for this analysis included hospital LOS as well as 30- and 90-day RR.

**Statistical analysis**

Patient characteristics and infectious complication outcomes were summarized within each BG trajectory group. Continuous variables were reported as median (range: min-max) and compared among groups using the Kruskal-Wallis test. Nominal variables were reported as n (%) and compared using Chi-square or Fisher’s exact test as appropriate. Median LOS was estimated by the Kaplan-Meier method with comparisons among groups made by the log-rank test. Cumulative incidence functions (CIF) were used to estimate time to 30-day readmission, 90-day readmission, and engraftment for each group, in a competing risk setting, with death treated as the competing event. Patients still alive without readmission or engraftment were censored at appropriate time or last day of follow-up. Patients without engraftment data following their first HCT who then received a second HCT were censored at their second HCT date (n = 1). Univariable hazard ratios (HR) for readmission and their corresponding 95% confidence intervals (CI) were estimated using Fine and Gray’s (1999) extension of Cox regression. Due to limited events, HR for engraftment was not estimable.

All statistical analyses described above were performed using SAS Version 9.4 (SAS Institute, Cary, NC). GBTM analysis was implemented with a SAS macro named PROC TRAJ^35^. Hypothesis testing was two-sided and conducted at the 5% level of significance.

## Results

**Patient characteristics**

745 patients were initially identified from the CIBMTR database, of whom 404 (54.2%) satisfied our inclusion and exclusion criteria and were the subjects in this study. Table 1 lists patient characteristics by pre-HCT BG trajectory group as described below. The median age at HCT was 53.0 years. The population was primarily male (57%) and racially mixed (39% Caucasian, 22% African American, 31% other), and was generally evenly divided between those who underwent allogeneic (56%) and autologous (44%) HCT. Among allogeneic HCT patients, the majority of donors were unrelated. The largest diagnosis category was “other,” which included diagnoses such as myelodysplasia and myelofibrosis. 

**BG trajectory groups**

21,169 glycemic measurements were analyzed in total, with 68% of measurements processed in the central laboratory and 32% from capillary point-of-care testing. 

**Figure 1:**
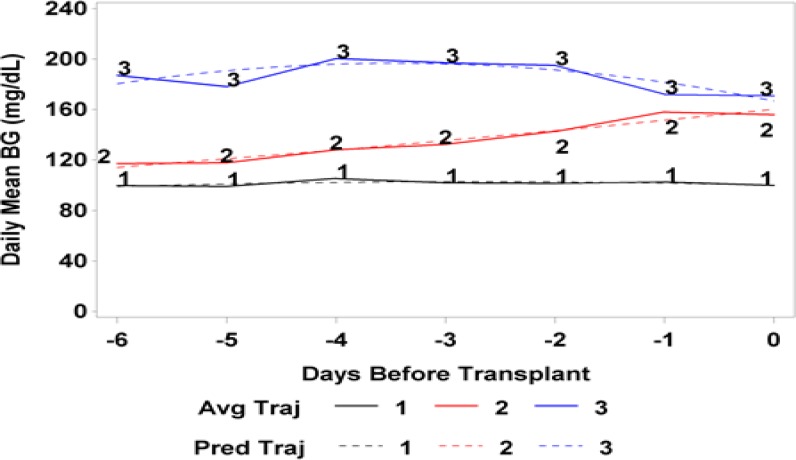
Daily Mean Blood Glucose Levels Beginning 6 Days Prior to Transplantation.

Three distinct BG trajectory groups were identified during the 7 days prior to HCT using the criteria mentioned previously (see Figure 1 for BG trajectories and Table 2 for fit statistics). Group 1 consisted of patients with consistently normal BG levels which remained below 120mg/dL during the week before HCT (n=317, 78.5%). Group 2 consisted of patients with varying BG levels that, on average, began at 120mg/dL at 7 days prior to HCT then increased steadily to approximately 160mg/dL by the day of HCT (n = 67, 16.6%). Group 3 consisted of patients with consistently high BG levels that averaged over 160mg/dL (n= 20, 4.9%). 

Groups 1 and 2 shared a similar age distribution ranging from infancy to adulthood, while group 3 was comprised only of adults. Group 1 was primarily Caucasian or African American in race and non-Hispanic in ethnicity. Group 2 was notable for the large percentage of patients undergoing an unrelated allogeneic HCT (43%), receiving immunosuppression (70%), and receiving steroids (57%). Group 3 was primarily male (80%), of Hispanic ethnicity (53%), and diabetic (90%). The percentage of endocrinology consultations varied significantly among the groups: 48% in Group 1, 54% in Group 2, and 85% in Group 3. 

**Table 1:** Patient Characteristics by Pre-HCT Blood Glucose Trajectory Group. Percentage composition does not include missing category

		**Pre-Transplant Blood Glucose Trajectory Group**			**P** 1
	**All** **N=404**	**1** **N=317**	**2** **N=67**	**3** **N=20**	
Age at Transplant Median (Min – Max)	53.0 (0.1 – 75.8)	52.9 (0.1 – 75.8)	52.9 (1.0 – 71.5)	56.6 (28.0 – 70.0)	0.3474^2^
BMI Median (Min – Max)	12 Missing 25.8 (12.5 – 85.8)	10 Missing 25.6 (12.5 – 85.8)	2 Missing 26.6 (15.8 – 57.2)	0 Missing 26.7 (18.7, 37.9)	0.0973^2^
Gender Female Male	175 (43%) 229 (57%)	143 (45%) 174 (55%)	28 (42%) 39 (58%)	4 (20%) 16 (80%)	0.0804
Race Caucasian African American Hispanic/Latino Asian/Native American/Other	14 Missing 150 (38%) 86 (22%) 32 (8%) 122 (31%)	10 Missing 126 (41%) 75 (24%) 22 (7%) 84 (27%)	4 Missing 19 (30%) 8 (13%) 8 (13%) 28 (44%)	0 Missing 5 (25%) 3 (15%) 2 (10%) 10 (50%)	0.0132*
Ethnicity Hispanic Non-Hispanic	138 Missing 71 (27%) 195 (73%)	109 Missing 49 (24%) 159 (76%)	24 Missing 14 (33%) 29 (67%)	5 Missing 8 (53%) 7 (47%)	0.0268*
Diabetic	92 (23%)	45 (14%)	29 (43%)	18 (90%)	<.0001*
GVHD	207 (51%)	160 (50%)	37 (55%)	10 (50%)	0.7739
ICU Stay	44 (11%)	32 (10%)	12 (18%)	0 (0%)	0.0497*
TPN Administered	96 (24%)	76 (24%)	14 (21%)	6 (30%)	0.6902
Steroids Administered	145 (36%)	102 (32%)	38 (57%)	5 (25%)	0.0004*
Immunosuppressants Administered	222 (55%)	166 (52%)	47 (70%)	9 (45%)	0.0192*
Endocrinology Consult	1 Missing 205 (51%)	1 Missing 152 (48%)	0 Missing 36 (54%)	0 Missing 17 (85%)	0.0039*
Bone Marrow Transplant Type Related Allogeneic Unrelated Allogeneic Autologous	1 Missing 94 (23%) 134 (33%) 175 (43%)	1 Missing 68 (22%) 100 (32%) 148 (47%)	0 Missing 21 (31%) 29 (43%) 17 (25%)	0 Missing 5 (25%) 5 (25%) 10 (50%)	0.0258*
Primary Diagnosis Multiple Myeloma Acute Myeloid Leukemia (AML) Acute Lymphocytic Leukemia (ALL) Hodgkin’s Lymphoma Non-Hodgkin’s Lymphoma Other	0 Missing 69 (17%) 81 (20%) 34 (8%) 32 (8%) 87 (22%) 101 (25%)	0 Missing 63 (20%) 57 (18%) 29 (9%) 23 (7%) 74 (23%) 71 (23%)	0 Missing 6 (9%) 20 (30%) 5 (8%) 7 (10%) 8 (12%) 21 (31%)	0 Missing 0 (0%) 4 (20%) 0 (0%) 2 (10%) 5 (25%) 9 (45%)	

1 Values of P were calculated by Chi-square test or Fisher’s exact test as appropriate, if not specified otherwise.

2 Values of P were calculated by Kruskal-Wallis test.

* : p < 0.05.

Avg = average. Traj = trajectory. BG = blood glucose

**Table 2:** Fit Statistics of Trajectory Models

	**Optimal number of groups for 1 Week Pre-Transplant Blood Glucose; N=404**			
	**Number of Groups**			
	**1**	**2**	**3**	**4**
Log likelihood	-12044.1	-11638.1	-11518.0	-11441.5
BIC	12056.1	11662.1	11554.0	11489.5
SSA-BIC	12045.5	11639.5	11519.4	11442.9
Entropy	–	11.7	33.1	34.0
Entropy (relative)	–	0.96	0.93	0.94
Two-group model	1	2		
1. *n*, 350 (87%)	**0.992**	0.031		
2. *n*, 54 (13%)	0.008	**0.969**		
Three-group model	1	2	3	
1. *n*, 317 (78%)	**0.976**	0.024	0	
2. *n*, 67 (17%)	0.062	**0.931**	0.007	
3. *n*, 20 (5%)	0	0.052	**0.948**	
Four-group model	1	2	3	4
1. *n*, 313 (78%)	**0.979**	0.015	0.006	0
2. *n*, 48 (12%)	0.053	**0.912**	0.025	0.01
3. *n*, 30 (7%)	0.054	0.021	**0.925**	0
4. *n*, 13 (3%)	0	0.007	0	**0.993**

Shaded cells are the selected model. Bold values represent the highest mean posterior probability values for each class within each model

**Clinical outcomes: infectious complications and time to engraftment**

Analysis of the association between pre-HCT BG trajectories and infectious complications, shown in Table 3, revealed that a significantly greater number of patients in Group 2 were diagnosed with pneumonia compared with either of the other groups (19% in Group 2 versus 7% in Group 1 and 10% in Group 3, p=.0054). No differences were noted in the probability of developing *C. difficile* infections, positive cultures, administration of antimicrobial agents, or occurrence of neutropenic fevers among the three groups. Group 2 also had the lowest percentage of patients who engrafted after HCT (85% in Group 2 versus 93% in Group 1 and 95% in Group 3) as well as the highest percentage of deaths (10% in Group 2 compared with 4% in Group 1 and 5% in Group 3). The median time-to-engraftment was 10 days for each group, and no significant differences were noted. 

**Table3:** Post-HCT Outcomes by Pre-HCT Blood Glucose Trajectory Group

	**Pre-Transplant Blood Glucose Trajectory Group**			**P**
	**1** **N=317**	**2** **N=67**	**3** **N=20**	
Infectious Complications				
Pneumonia	21 (7%)	13 (19%)	2 (10%)	0.0054*^3^
Antibiotics Administered^1^	229 (72%)	55 (82%)	17 (85%)	0.1467^3^
Clostridium Difficile (C.difficile)	22 (7%)	4 (6%)	1 (5%)	1.0000^3^
Neutropenia	295 (93%)	63 (94%)	19 (95%)	1.0000^3^
Positive culture of microorganism (N=72)^2^	N = 59 38 (64%)	N = 11 7 (64%)	N = 2 1 (50%)	1.0000^3^
Positive culture of microorganism (N=404)	38 (64%)	7 (64%)	1 (50%)	0.7533^3^
Time to Engraftment				
Engraftment Status Engrafted No Engraftment (Censored) Death	1 Missing 295 (93%) 7 (2%) 14 (4%)	0 Missing 57 (85%) 3 (4%) 7 (10%)	0 Missing 19 (95%) 0 (0%) 1 (5%)	
Median Days to Engraftment	10	10	10	
Probability of Engraftment within 7 days of Transplant [95% CI]	0.6% [0.2%-2.5%]	0.8% [0.2%-2.5%]	0.9% [0.3%-2.8%]	
Probability of Engraftment within 14 days of Transplant [95% CI]	78.2% [74.5%-82.1%]	80.6% [74.8%-86.8%]	83.5% [75.6%-92.1%]	
Probability of Engraftment within 28 days of Transplant [95% CI]	95.1% [92.5%-97.8%]	96.1% [93.2%-99.1%]	97.2% [94.1%-100%]	
Health Resource Utilization				
Length of Stay	23 (19-36)^4^	26 (20-48)^4^	22 (18-30)^4^	0.0157*^5^
Number at risk for Readmission^6^	**N=295** 6	**N=62** 6	**N=20** 6	
30 Day Readmission Readmitted Not Readmitted (Censored) Death Hazard Ratio [95% CI]	90 (31%) 200 (68%) 5 (2%) Reference	20 (32%) 41 (66%) 1 (2%) 1.04 [0.65, 1.66]	5 (25%) 15 (75%) 0 (0%) 0.82 [0.33, 2.07]	0.9000^5^
90 Day Readmission Readmitted Not Readmitted (Censored) Death Hazard Ratio [95% CI]	126 (43%) 143 (48%) 26 (9%) Reference	31 (50%) 25 (40%) 6 (10%) 1.23 [0.83, 1.80]	7 (35%) 11 (55%) 2 (10%) 0.80 [0.37, 1.72]	0.4586^5^

1 Cefepime or Vancomycin Administered during hospitalization.

2 Only 72 patients in the final cohort have culture information available.

3 A value of P computed from Fisher’s Exact Test.

4 Median (Q1-Q3) from Competing risk model**. **

5 A value of P computed from Competing Risk Model**. **

6 Patients that died prior to discharge are excluded from the analyses of readmission as they were never at risk

**Quality Outcomes: Health Resource Utilization**

The analysis of the association between pre-HCT BG trajectories and health resource utilization also shown in Table 3, indicated that those in Group 2 (varying glucose levels) had a statistically significant longer median hospital LOS than did those in either of the other groups: Group 1-23 days, Group 2-26 days, Group 3-22 days (p = .0157). The risk of 90-day readmission was 23% greater in Group 2 relative to Group 1 (HR [95% CI]: 1.23 [0.83-1.80] and 20% less in Group 3 relative to Group 1 (HR [95% CI]: 0.80 [0.37-1.72], although this was not statistically significant. Similar HRs were observed for 30-day readmission rates. 

## Discussion

 The apparent link between malglycemia and outcomes after HCT has been the focus of an increasing number of studies in recent years. Regardless, further investigation is necessary to better elucidate the nature of this association to inform clinical practice. Our data adds to the existing body of literature in this realm by confirming our initial hypothesis that pre-HCT glucose trends can serve as a potential prognostic biomarker for post-HCT outcomes, both clinically and in terms of health resource utilization. 

With regards to the clinical outcomes, we found a significant increase in the diagnosis of post-HCT pneumonia among patients with varying glucose levels compared to those with stable levels in either normal or elevated ranges. Although no differences were found in terms of the other infectious complications, our data corroborates the previously established association between serum BG levels and the incidence of infection in this particular population. 

Based on our data, pre-HCT BG trends also appear to have an association with health resource utilization. We studied this effect as it relates to three metrics that are widely used to measure clinical quality, namely hospital LOS and 30-day and 90-day RR. Although no differences were found with regards to RR, our data do demonstrate a significant increase in LOS among patients with varying glucose levels compared to patients with stable levels. Aside from the important sequelae for individual patients and their families (and resultant patient satisfaction scores), this increased LOS has significant implications both at the institutional level (in terms of hospital reimbursement, throughput, and efficiency) and at the broader macroeconomic level (in terms of total healthcare spending). 

An important and surprising finding was that the significant associations were found only within those patients with varying BG levels (Group 2). Given the extensive data discussed above regarding the link between hyperglycemia and adverse outcomes, we expected that the group with the highest BG levels (Group 3) would have the most significant differences in terms of complications and resource utilization. A possible explanation is that the Group 3 patients, who were hyperglycemic from the beginning, were perhaps more likely to stand out to clinicians on account of their abnormal lab values and thus may have been more likely to receive appropriate care and closer monitoring during their HCT. The fact that 90% of the patients in this group had pre-existing diabetes mellitus also supports this theory, and likely led to the much higher percentage of inpatient endocrinology consultations in this cohort. Along the same lines, patients in Group 2 began with mostly normal BG levels and thus were perhaps less likely to initially garner attention in this way, despite the fact that over the ensuing days their BG levels slowly increased. This under-recognition may have allowed their gradually worsening hyperglycemia to go unnoticed, thereby potentially increasing the risk of post-HCT complications. It remains to be determined if varying glucose levels are perhaps a clinical biomarker for adverse outcomes, or if these fluctuations, in addition to steroids and immunosuppression, create conditions conducive to adverse outcomes in HCT.

Our study has several limitations which should be considered when designing further studies in this area. The largest limitation is the retrospective, single-center design, as a multi-center prospective study would be preferred. In addition, we only looked at BG levels in the 7 days prior to HCT, which may be subject to more variation and thus may not accurately capture broader glycemic trends for each patient. In future studies, it would be interesting to extend BG measurements to 1-3 months prior to HCT, or to use hemoglobin A1c readings, though this may be prone to variations depending on stability of hemoglobin levels. Furthermore, although our outcomes included health resource utilization, a quantitative analysis based on the cost implications of an increased average LOS may be more impactful and would be a logical next step for a related study. 

## CONCLUSION

 Despite these limitations, this work has already changed clinical practice at our institution. We have partnered with our endocrinologists to discuss ways to enhance the care of our HCT patients. Among other things, this discussion led to a protocol in which hemoglobin A1c levels are checked prior to HCT to facilitate timely outpatient endocrinology consultation. Using our retrospective findings as a foundation, we aspire to formulate a multidisciplinary malglycemia management program for HCT patients as part of a prospective cohort study to explore whether such a dedicated intervention has an effect on patient outcomes in both the clinical and quality realms. We look forward to implementing this program and sharing our findings in future work
